# Understanding Acceptability and Willingness-to-pay for a C-reactive Protein Point-of-care Testing Service to Improve Antibiotic Dispensing for Respiratory Infections in Vietnamese Pharmacies: A Mixed-methods Study

**DOI:** 10.1093/ofid/ofae445

**Published:** 2024-08-02

**Authors:** Nam Vinh Nguyen, Nga Thi Thuy Do, Huong Thi Lan Vu, Phuong Bich Bui, Thai Quang Pham, Vinh Thanh Khuong, Anh Tuan Lai, H Rogier van Doorn, Sonia O Lewycka

**Affiliations:** Oxford University Clinical Research Unit, Hanoi, Vietnam; Health Economic Research Centre, Nuffield Department of Population Health, University of Oxford, Oxford, UK; Oxford University Clinical Research Unit, Hanoi, Vietnam; Oxford University Clinical Research Unit, Hanoi, Vietnam; Oxford University Clinical Research Unit, Hanoi, Vietnam; National Institute of Hygiene and Epidemiology, Hanoi, Vietnam; Nam Dinh Department of Health, Vietnam; Nam Dinh Center for Disease Control and Prevention; Oxford University Clinical Research Unit, Hanoi, Vietnam; Nuffield Department of Clinical Medicine, University of Oxford, Oxford, UK; Oxford University Clinical Research Unit, Hanoi, Vietnam; Nuffield Department of Clinical Medicine, University of Oxford, Oxford, UK

**Keywords:** antimicrobial resistance, antimicrobial stewardship, community pharmacy, low and middle income countries (LMICs), point-of-care testing

## Abstract

**Background:**

Pharmacies are popular first points of contact for mild infections in the community. Pharmacy services in many countries have expanded to include vaccines and point-of-care tests. In low- and middle-income countries such as Vietnam, poor enforcement of regulations results in substantial volumes of over-the-counter antibiotic sales. Point-of-care tests could provide an economically viable way to reduce antibiotic sales, while still satisfying customer demand for convenient healthcare. C-reactive protein point-of-care testing (CRP-POCT) can reduce antibiotic prescribing for respiratory illness in primary care. Here, we explore the acceptability and feasibility of implementing CRP-POCT in pharmacies in Vietnam.

**Methods:**

We conducted a mixed-methods study between April and June 2021. A customer exit survey with 520 participants seeking acute respiratory infection treatment at 25 pharmacies evaluated acceptability and willingness-to-pay (WTP) for CRP-POCT and post-service satisfaction. Factors driving customers” acceptance and WTP were explored through mixed-effects multivariable regression. Three focus group discussions with customers (20 participants) and 12 in-depth interviews with pharmacists and other stakeholders were conducted and analyzed thematically.

**Results:**

Antibiotics were sold to 81.4% of patients with CRP levels <10 mg/L (antibiotics not recommended). A total of 96.5% of customers who experienced CRP-POCT supported its future introduction at pharmacies. Patients with antibiotic transactions (adjusted odds ratio [aOR], 2.25; 95% confidence interval [CI], 1.13–4.48) and those suffering acute respiratory infection symptoms for more than 3 days (aOR, 2.10; 95% CI, 1.08–4.08) were more likely to accept CRP-POCT, whereas customers visiting for children (aOR, 0.20; 95% CI, .10–.54) and those with preference for antibiotic treatment (aOR, 0.45; 95% CI, 0.23–0.89) were less likely to accept CRP-POCT. A total of 78.3% (95% CI, 74.8–81.7) of customers were willing to pay for CRP-POCT, with a mean cost of US$2.4 (±1.1). Customer's income and cost of total drug treatment were associated with increased WTP. Enablers for implementing CRP-POCT included customers’ and pharmacists’ perceived benefits of CRP-POCT, and the impact of COVID-19 on perceptions of POCT. Perceived challenges for implementation included the additional burden of service provision, lack of an enabling policy environment, and potential risks for customers.

**Conclusions:**

Implementing CRP-POCT at pharmacies is a feasible and well-accepted strategy to tackle the overuse of antibiotics in the community, with appeal for both supply and demand sides. Creating an enabling policy environment for its implementation, and transparent discussion of values and risks would be key for its successful implementation.

Vietnam has amongst the highest levels of antibiotic use and antibiotic resistance in Asia [1, 2]. Economic reforms during the 1980s and 1990s transformed Vietnam from a low-income to a lower to middle-income country (LMIC) by 2013. These reforms also facilitated an increase in the number of private drug retailers [1]. The drug retail system includes more than 60 000 pharmacies and is Vietnam's most popular first point of contact for primary care. A national survey on pharmacy sales of antibiotics in 2016 showed that nearly 30% of drug sale transactions at pharmacies included purchasing antibiotics [3]. Although Vietnamese regulations require that antibiotics be sold only with a prescription, regulations are not enforced, and approximately 90% of pharmacy antibiotic transactions are made without a prescription [4].

Minimizing inappropriate sales of antibiotics at pharmacies is vital for preventing and controlling the spread of antimicrobial resistance (AMR), but pharmacy interventions tackling this issue in LMICs are limited [5]. Most interventions focus on education and enforcement of regulatory barriers [6, 7]. However, economic drivers and widespread misconceptions about the role of antibiotics also need to be addressed. Point-of-care testing (POCT) services to guide antibiotic treatment are promising because they can compensate for lost profits from not selling antibiotics while still satisfying patients’ demands for convenient healthcare and providing visual evidence to improve understanding of when antibiotics are unnecessary [8].

C-reactive protein (CRP), an acute marker of inflammation, is a biomarker that can be used to guide antibiotic treatment in patients with acute respiratory infections (ARI), the most common reason for antibiotic supply in the community [9, 10]. Levels of CRP increase in response to inflammation, infection, or injury and are generally raised in bacterial infections, but not in viral infections. CRP point-of-care testing (CRP-POCT) to guide antibiotic use in primary care has been widely studied in high-income countries and found to reduce inappropriate prescribing of antibiotics [11, 12]. In Vietnam, 2 randomized controlled trials showed that the use of CRP-POCT in primary care settings significantly reduced antibiotic prescribing in patients with nonsevere ARIs without compromising their recovery or inducing serious adverse events [13, 14]. There is also evidence that CRP testing may be useful in low-resource settings to improve rational antibiotic use for other conditions, such as acute febrile illness, but the positive predictive value might not be sufficient to allow it to be used alone as a single tool for these conditions [15].

The potential benefits of implementing CRP-POCT at pharmacies have been studied in several countries. A pilot study in a rural pharmacy in North Staffordshire, UK, evaluated the potential use of CRP-POCT to reduce demand for general practitioner services for respiratory tract infections. Of patients who received CRP-POCT at pharmacies, 95% reported that if the service had not been conducted, they would have otherwise visited the general practitioner and expected to be prescribed antibiotics [16]. A study in Western Australia showed that 50.9% of pharmacy customers with ARI changed their perceptions regarding their need for antibiotics after receiving CRP test results [17]. A parallel cluster randomized controlled trial in Nigeria showed that using CRP-POCT in pharmacies reduced antibiotic dispensing for ARIs by 15.7% [18].

These findings suggest that the implementation of CRP-POCT at pharmacies has the potential as an intervention to reduce community antibiotic use. However, the lack of POCT services currently available at pharmacies in Vietnam means that evidence on acceptability and willingness-to-pay (WTP) for such a service is required to guide policymakers, alongside information about potential enablers and challenges.

This study investigates the acceptability and feasibility of introducing CRP-POCT to guide referral for antibiotic treatment for ARIs at pharmacies in northern Vietnam.

## METHODS

### Study Design and Settings

We conducted a mixed-methods study using an explanatory sequential design. A customer exit survey was conducted to estimate acceptance and WTP for CRP-POCT and the factors driving their decisions. We collected qualitative data through focus group discussions (FGD) and in-depth interviews (IDI) to gain further insight into these factors and other aspects not captured in the quantitative component. (Supplementary Document 1 provides a diagram explaining our study design.) Quantitative data and most qualitative data (all FGDs and 9 of 12 IDIs) were collected in Nam Dinh City, an urban area located 90 km southeast of Hanoi, where neither malaria nor dengue are endemic. We also conducted 3 IDIs in Hanoi to provide perspectives from a larger urban setting.

### Sampling and Participant Recruitment

We used a multistage sampling procedure to select pharmacies and participants for the exit survey. We obtained a list of the pharmacies registered with the local health authority, contacted pharmacy owners via phone, and randomly selected 25 pharmacies from those willing to participate. A sample size of 520 customer exit interviews would be needed to estimate both the proportion accepting CRP-POCT and the mean WTP. We estimated that half of them (n = 260) would be willing to pay for CRP-POCT and would be offered the test. The sample size of 260 was also sufficient to estimate the proportion of unnecessary antibiotic transactions (Supplementary Document 2). To reach the required sample sizes, interviewers approached customers after completing a medication transaction and screened against the eligibility criteria to recruit 20 to 21 individuals at each pharmacy. They included customers aged 18 years and older seeking treatment for mild ARI for themselves or relatives (Supplementary Document 3 provides details of eligibility criteria). Limiting the number of customers recruited in each pharmacy to around 20 also allowed our study team to control for the risk of accidentally approaching the same patient more than once, which could lead to measurement errors.

In the qualitative component, we used purposive sampling to select respondents representing stakeholders who could provide rich insights to inform potential CRP-POCT implementation in the future [19]. We recruited 20 customers into FGDs and 6 pharmacists into IDIs from those who participated in the exit survey. IDIs, with other participants in Nam Dinh City, were selected by local study staff to provide relevant insights to inform potential implementation of CRP-POCT services in the future. They included 1 general practitioner, 1 pediatrician, and 1 local health authority official. For perspectives from a larger urban area, we also selected 2 pharmacists and 1 medical device wholesaler for IDIs in Hanoi.

### Data Collection Procedures

We collected data between April and June 2021. There were localized COVID-19 outbreaks during this period, but no community transmission in the study area.

Quantitative data were collected through face-to-face exit interviews using a structured questionnaire (Supplementary dDocument 4). The interviewers were local preventive medicine physicians and nurses, trained over 3 days in interviewing skills, performing CRP-POCT, and Good Clinical Practice. For each customer, an interviewer first screened against eligibility criteria and interviewed eligible, consenting participants about demographic information, reasons for their visit to the pharmacy, and treatment transactions. Then, they described CRP-POCT and asked if the participant accepted it as an out-of-pocket service to improve antibiotic targeting. The participants who accepted were invited to a bidding game to elicit their WTP and randomly allocated to 1 of 4 different starting prices (Supplementary Document 5 describes how a bidding game was implemented). CRP-POCT was offered to the first 10 to 11 participants among those accepting the CRP-POCT at each pharmacy. We reported CRP test results to customers, asked about their satisfaction with the service, and how the results would influence the use of any antibiotics they had already bought. Each customer, whether they were willing to use the CRP-POCT or agreed to be tested or not, was paid VND100,000 (∼US$4) as compensation for their time (approximately 45 minutes). We treated different groups of participants equally to avoid interview bias.

We conducted the data collection in 3 different rounds: 5 pharmacies in the first round, 10 pharmacies in the second round, and 10 pharmacies in the final round. Completed data collection forms were regularly sent to a study coordinator (B. B. P.) to check for completeness and other errors and reported to a study investigator (N. V. N.). The quality of the data collection was checked by 2 coordinators (V. T. K. and B. B. P.) and 1 investigator (N. V. N.). Regular meetings between data collectors, study coordinators, and study investigators were arranged during data collection to discuss issues that occurred and how to address them.

We used the Actim CRP Rapid Test from Medix Biochemica (Espoo, Finland), costing 2.5€ ($USD2.7) per test [20]. The test is a simple lateral flow device with minimal training requirements that uses capillary blood obtained through a finger/heel puncture. The test provides a semiquantitative indication in under 5 minutes of whether CRP concentrations are <10 mg/L, between 10 and 40 mg/L, between 40 and 80 mg/L, or >80 mg/L. We did not use the CRP test results to guide treatment because they were conducted after customers had already transacted with the pharmacist, but we determined the appropriateness of any antibiotic treatment given. In the absence of danger signs of severity, guidance CRP levels were: below 10 mg/L = no antibiotics recommended; between 10-40 mg/L = antibiotics unlikely to be needed but patients would be advised to seek further medical guidance and antibiotics could be considered in cases of high clinical concern (including those who were systemically very unwell, those with a preexisting comorbidity, those currently using oral corticosteroids or those with a history of congestive heart failure); above 40 mg/L = antibiotics recommended and patients advised to seek further medical guidance [20]. The test was used for research use only, and all test procedures were conducted and interpreted by medical professionals for the specific purposes of our study.

Qualitative data were collected through face-to-face, audio-recorded interviews using interview guides (Supplementary Document 6). Participants of FGDs were grouped into 3 groups according to their acceptance of the CRP-POCT service: those who accepted the service (10 participants), those who refused the service (4 participants), and 1 mixed group (6 participants). The homogenous groupings enabled respondents to share their opinions comfortably, whereas the mixed group allowed respondents to argue against opposing opinions.

### Data Analysis

The quantitative analysis used descriptive statistics to investigate the feasibility of implementing CRP-POCT to support ARI management at community pharmacies. Primary outcomes included the proportion of customers accepting CRP-POCT and mean WTP. Reasons for refusing the service were also reported and ranked by the frequency of answers.

Mean WTP was estimated only among respondents who were willing to pay for the service. WTP and other monetary data were collected in Vietnamese Dong and converted to US dollars using the 2021 World Bank exchange rate: USD$1 = VND23,159.78 (https://data.worldbank.org/indicator/PA.NUS.FCRF). We reported WTP corresponding to different initial bidding prices to see the impact of starting point bias.

Determinants of accepting CRP-POCT were explored through mixed-effects logistic regression. Determinants of WTP for CRP-POCT were explored through mixed-effects linear regression. Variables included in the models were identified through a literature review of previously published studies [21, 22]. In both models, pharmacy ID was included as a random effect to consider clustering effects by pharmacy. Fixed effects included age, sex, educational class, income group, customer's perception of antibiotic role, antibiotic transaction, child patient, prior visit to doctor, duration of illness, type of pharmacy, cost of treatments, and degree of satisfaction with pharmacy service. The initial bid price was also included in the model as a fixed effect to investigate the impact of starting point bias on WTP.

To investigate the appropriateness of antibiotic use, we calculated the proportion of patients with antibiotic transactions for each CRP level, among the 260 tested participants. Those with CRP <40 mg/L were considered inappropriate—antibiotic treatment would not have been recommended or was unlikely to be needed. We also reported the proportions of customers supporting the introduction of CRP-POCT at pharmacies, trusting in pharmacists’ capacity to undertake the test, and confirming they would change their behavior toward antibiotic use following the test results.

In the qualitative analysis, we performed a thematic analysis using inductive coding [23]. The analysis consisted of 5 steps: (1) familiarizing with data, (2) generating initial codes from an inductive and data-driven process, (3) searching for themes and subthemes and extracting data related to them, (4) reviewing themes at the level of the coded data extracts and at the level of the entire dataset, and (5) defining themes and creating a thematic map illustrating the relationships between sub-themes and themes. One person (N. V. N.) performed the data analysis, and the thematic map with corresponding quotations was presented internally among the coauthors and externally with 2 health service researchers to gain additional perspectives.

R version 3.3.3 was used for the quantitative statistical analysis, and QSR International's NVivo 11 Software was used for the qualitative statistical analysis.

### Ethical Considerations

The study was reviewed and approved by the National Institute of Hygiene and Epidemiology Institutional Review Board in Biomedical Research (IRB-VN01057) and Oxford Tropical Research Ethics Committee, University of Oxford (Reference 529-19). All patients/participants provided their written informed consent to participate in this study.

## RESULTS

### Characteristics of Participants in Exit Surveys

Of 55 pharmacies that responded to our invitation to join the study, 42 (76.4%) accepted it. Among 13 pharmacy owners who refused to join our study, 8 were afraid of the risks of COVID-19 transmission because of long interactions between interviewers and customers, and 5 did not give a reason. Twenty-five pharmacies were randomly selected from these 42 pharmacies, where we approached a total of 577 customers seeking treatment for ARIs and invited them to join the study. A total of 528 (91.5%) accepted. The main reason for refusing was lack of time (87.8%). The study interviewers screened these 528 patients against the eligibility criteria. Eight were not eligible and 520 were recruited to the study (Supplementary Document 7).

Of 520 surveys, 40.6% (211/520) were conducted at privately owned pharmacies, 34.8% (181/520) at chain pharmacies, and 24.6% (128/520) at retail pharmacies in hospitals/clinics. The median age of participants was 41.5 years (interquartile range, 31–58 years); 71.7% were female, and nearly half had a university/college or higher (49.5%) degree. Participant's average monthly household per capita income was USD$264.4 (± 129.1). A total of 78.3% believed that antibiotic treatment was necessary in their situation and 79.4% bought antibiotics. Only 8.3% saw a doctor before their pharmacy visit. The majority of customers visited the pharmacy to seek treatment for adult patients (90.8%) and for an illness lasting equal to or less than 3 days (74.6%). The mean cost of total drug treatment was USD$3.6 (± 4.0). Almost all customers were either very satisfied (81.8%) or satisfied (18.1%) with pharmacy services in the region (Table 1).

**Table 1.** Baseline Characteristics of Participants

	Number Of Customers (n = 520)	
Customer's age, median [1st, 3rd quartiles] (y)	41.5	(31–58)
Female sex (n [%])	373	(71.7)
Highest education (n [%])		
Preliminary school	143	(27.5)
High school	108	(20.8)
University/college and higher degree	261	(50.2)
Unclassified (prefer not to say)	8	(1.5)
Mean (± SD) household monthly income per person^a^ (2021 $USD)	260.5	(± 127.6)
Income group classified by average household monthly income per person (n [%])		
< VND 3 000 000 (< $USD129.5)	82	(15.8)
VND 3 000 000–5 000 000 ($USD129.5–215.9)	205	(39.4)
> VND 5 000 000 (>$USD215.9)	229	(44.0)
Unclassified (prefer not to say)	4	(0.8)
Customer's perception on the role of antibiotics to the patient's condition		
Antibiotic treatment is needed	407	(78.3)
Antibiotic treatment is not needed	113	(21.7)
Antibiotic transaction		
Yes	413	(79.4)
No	107	(19.6)
Visiting pharmacy to seek treatments for (n [%])		
Adults	472	(90.8)
Childs	48	(9.2)
Seeing a doctor prior pharmacy visit (n [%])		
Yes	43	(8.3)
No	477	(91.7)
Duration of illness (n [%])		
±3 d	388	(74.6)
>3 d	132	(25.4)
Mean (± SD) cost of total drug treatment ($USD)	3.6	(4.0)
Type of pharmacy visited		
Privately owned (neighborhood) pharmacy	211	(40.6)
Retailed pharmacy chain	181	(34.8)
In-hospital/in-clinic retailed pharmacy	128	(24.6)
Degree of satisfaction to pharmacy service in the region^b^, (n [%])		
Very satisfied	422	(81.8)
Satisfied	94	(18.1)
Neutral	4	(0.8)
Dissatisfied	0	(0)
Very dissatisfied	0	(0)

^a^Household monthly income per person was calculated by dividing their household's total income from by the number of individuals who belong to her or his household.

^b^Degree of satisfaction with pharmacy services of a consumer was extrapolated from their answer to a question using 10-point Likert scale as following: very satisfied (9–10 points), satisfied (7–8 points), neutral (5–6 points), dissatisfied (3–4 points), and dissatisfied (1–2 points).

### Acceptability and Willingness-to-pay

**Figure 1.**
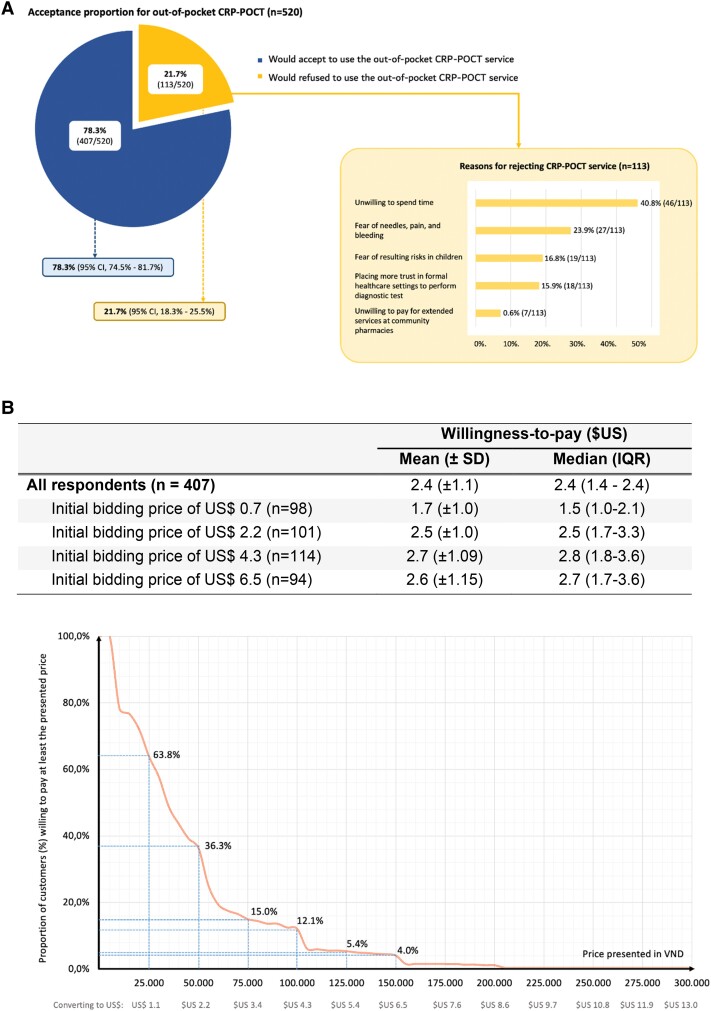
(*A*) Acceptability of CRP POCT service for patients with ARIs at pharmacies and the reasons leading to rejection (our interviewers asked the participants rejecting the service by an open-ended question. The answers were subsequently labeled and categorized in the themes shown). (*B*) Willingness-to-pay for CRP-POCT service and the relationship between price (x axis) and the proportion of customers willing to pay at least the presented price (y axis) in VND currency.

A total of 407 of 520 participants (78.3%; 95% confidence interval [CI], 74.5–81.7]) would be willing to pay for CRP-POCT at a pharmacy. For the 113 people who refused the service, the most common reason was the time constraint (40.8%), followed by fear of needles, pain, and bleeding (23.9%) (Figure 1*A*). Of those who were willing to pay, 260 were offered an actual CRP-POCT and 147 were provided with a descriptive scenario. Mean WTP among the customers accepting CRP-POCT as an out-of-pocket service was $USD2.4 (± 1.1), of whom 36.5% were willing to pay the price higher than $USD2.2 (VN 50 000), and 5.4% were willing to pay at a price higher than $USD5.4 (VND 150 000). The mean WTP of low initial bidding prices was lower than that of high initial bidding prices, showing a potential impact of starting point bias (Figure 1*B*).

### Determinants of Acceptability and Willingness-to-pay

Our results showed that visits with antibiotic transactions (adjusted odds ratio [aOR], 2.25; 95% CI, 1.13–4.48; *P* = .020) and patients suffering ARI symptoms for more than 3 days (aOR, 2.10; 95% CI, 1.08–4.08; *P* = .027) were associated with higher acceptance of CRP-POCT. Meanwhile, visits for children (aOR, 0.20; 95% CI, .10–.54; *P* < .001) and customers with a preference for antibiotic treatment (aOR, 0.45; 95%, CI, 0.23–0.89; *P* = .022) were associated with lower acceptance (Table 2).

**Table 2.** Factors Driving the Acceptability and the Willingness-to-pay for the CRP-POCT Service at Community Pharmacies

Variables	Acceptability^a^			Willingness-to-pay^b^	
	Acceptance rate (%, n/N)	Adjusted odd ratio (95%CI)	*P*-value	β (SE)	*P*-value
Age	-	0.99 (.97–1.00)	.169	−0.004 (0.002)	.075^†^
Sex					
Male	83.0 (122/147)	(ref)		(ref)	.504
Female	76.4 (285/373)	.65 (.37–1.15)	.140	−0.025 (0.058)	
Education class					
Preliminary school	78.3 (112/143)	(ref)		(ref)	…
High school	81.5 (88/108)	1.03 (.49–2.18)	.942	.060 (.081)	.537
University/college or higher degree	79.3 (207/261)	1.37 (.67–2.83)	.384	.044 (.082)	.471
Income groups					
< VND 3 000 000 (< $USD129.5)	74.3 (61/82)	(ref)		(ref)	
VND 3 000 000–5 000 000 ($USD129.5–259.1)	87.8 (180/205)	1.02 (.48–2.14)	.968	0.266 (0.081)	.001**
> VND 5 000 000 (> $USD259.1)	79.3 (182/229)	1.15 (.55–2.40)	.704	0.232 (0.079)	.004**
Customer's perception on the necessity of antibiotic treatment					
Antibiotic treatment is not needed	82.0 (118/144)	(ref)		(ref)	
Antibiotic treatment is needed	76.2 (289/376)	.45 (.23–.89)	.022*	0.004 (0.067)	.950
Antibiotic transaction					
Yes	80.4 (332/413)	2.25 (1.13–4.48)	.020*	0.124 (0.075)	.100
No	70.1 (75/107)	(ref)		(ref)	
Visiting pharmacy to seek treatments for					
Child	54.5 (22/44)	.23 (.10–.54)	<.001***	0.109 (0.118)	.353
Adult	80.5 (383/476)	(ref)		(ref)	
Seeing a doctor prior pharmacy visit					
Yes	79.0 (34/43)	1.19 (.36–3.91)	.781	−0.064 (0.144)	.657
No	83.2 (397/477)	(ref)		(ref)	
Duration of illness					
≤ 3 d	73.7 (286/388)	(ref)		(ref)	
>3 d	84.1 (111/132)	2.10 (1.08–4.08)	.029*	0.029 (0.070)	.667
Cost of total drug treatment	-	1.00 (.95–1.07)	.816	0.134 (0.061)	.027*
Type of pharmacy visited					
Privately owned pharmacy	75.2 (159/211)	(ref)		(ref)	…
Retailed pharmacy chain	81.2 (147/181)	.51 (.15–1.78)	.292	0.156 (0.127)	.230
In-hospital/in-clinic pharmacy	83.6 (107/128)	.40 (.11–1.43)	.155	−0.116 (0.136)	.400
Degree of satisfaction to pharmacy service	-	.77 (.37–1.61)	.507	0.002 (0.042)	.954
Initial bidding price	-	-	-	0.059 (0.120)	<.001***

β, beta-coefficients; SE, standard error.

^a^A mixed effects logistic regression model with odd ratios (ORs). Fixed effects included age, sex, education class, income groups, customer's perception on the role of antibiotic treatment, status of purchasing antibiotics, child patients, status of seeing doctor at a prior pharmacy visit, duration of illness, type of pharmacy, cost of total drug treatment. and degree of satisfaction to pharmacy service. Random effect included pharmacy ID.

^b^A mixed effects linear regression model with participants’ WTP transformed into log values. The log transformation was made because the distributions of WTP values were right skewed while the distributions of their log transformed values were more normalized. Fixed effects included age, sex, education class, income groups, customer's perception on the role of antibiotic treatment, status of purchasing antibiotics, child patients, status of seeing doctor prior pharmacy visit, duration of illness, type of pharmacy, cost of total drug treatment, degree of satisfaction to pharmacy service, and initial bidding price (to consider starting point bias). Random effect included pharmacy ID.

***P < .001, **P < .01, *P < .05, ^†^*P* < .10.

We also found that customers’ income and the cost of the total drug treatment were associated with increased customers’ WTP for CRP-POCT (Table 3). Varied initial bidding prices were associated with varied WTP, showing the impact of starting point bias. We examined the robustness of the main model by comparing results with another model that used the original values of WTP instead of transforming them into log values (Supplementary Document 8). Similar results between the 2 models show robustness of the findings.

**Table 3.** C-reative Protein Test Results of the Total Patients With Acute Respiratory Infection Symptoms who Were Offered CRP

	Total Patients Undertook CRP POCT (N = 260)	Patients With Antibiotic Transactions (N = 213)	Patients Without Antibiotic Transactions (N = 47)
CRP < 10 mg/L, % (n/N)	95.0 (247/260)	94.4 (201/213)	97.9 (46/47)
10 mg/L ≤ CRP < 40 mg/L, % (n/N)	5.0 (13/260)	5.6 (12/213)	2.1 (1/47)
CRP ≤ 40 mg/L, % (n/N)	0 (0/260)	0 (0/213)	0 (0/47)

### Unnecessary Antibiotic Transactions and Post-test Perceptions

Unnecessary sales/treatment with antibiotics was very common. Among the 213 CRP-tested patients with antibiotic transactions, 94.4% had CRP levels below 10 mg/L (antibiotic treatment should not have been provided), and 5.6% had CRP levels between 10 and 40 mg/L (antibiotic treatment was unlikely to be needed) (Table 3). No patient had CRP levels above 40 mg/L (antibiotic treatment was necessary). Of the 260 customers receiving CRP-POCT, 96.5% agreed that the service should be offered at pharmacies in the future, 97.7% maintained their pretesting WTP, and 81.2% trusted in pharmacists’ capacity to undertake the testing procedure. However, only 27.7% of the CRP-tested patients with antibiotic transactions said they would change their decision on antibiotic use following the test result (Table 4).

**Table 4.** Customer's Perceptions After Experiencing the CRP-POCT Service

	Total Patients Offered CRP-POCT (N = 260)
Do you think that this CRP-POCT service should be offered at community pharmacies?	
Yes	96.5 (351/260)
No	3.5 (9/260)
Would you like to change your previous decision on WTP after experiencing CRP-POCT?	
Yes—increased WTP	0 (0/260)
Yes—decreased WTP	2.3 (6/260)
No—remained WTP	97.7 (254/260)
Do you think pharmacists can undertake the test?	
Yes	81.2 (211/260)
No	18.8 (49/260)
Would you change your decision on antibiotic purchase following the recommendation of the testing result?	
Yes	27.7 (59/213)
No	3.3 (7/213)
Unsure	69.0 (147/213)

### Enablers and Challenges for the Implementation of CRP-POCT at Pharmacies

**Figure 2.**
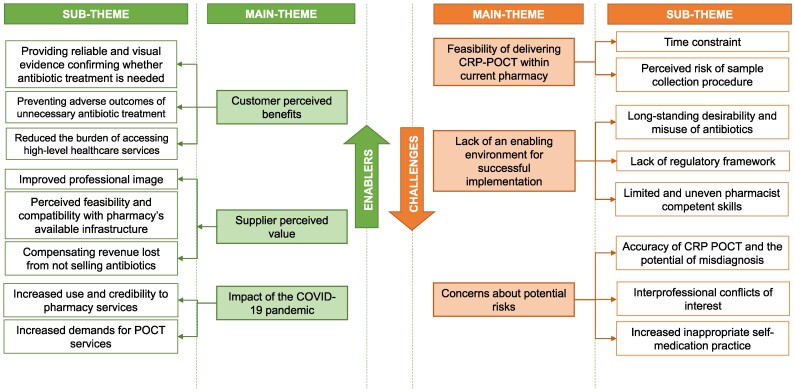
Themes and subthemes represented as enablers and challenges for implementation of CRP-POCT at community pharmacies in support of appropriate.

Figure 2 shows the main themes and sub-themes that emerged from our qualitative data analysis, representing enablers and challenges for CRP-POCT implementation. Verbatim quotes from the panel to illustrate the themes and sub-themes on enablers and challenges for CRP-POCT implementation in pharmacies are presented in Box 1.

Box 1. **Verbatim Quotes from the Panel to Illustrate the Themes and Sub-themes on Enablers and Challenges for CRP-POCT Implementation in Pharmacies**

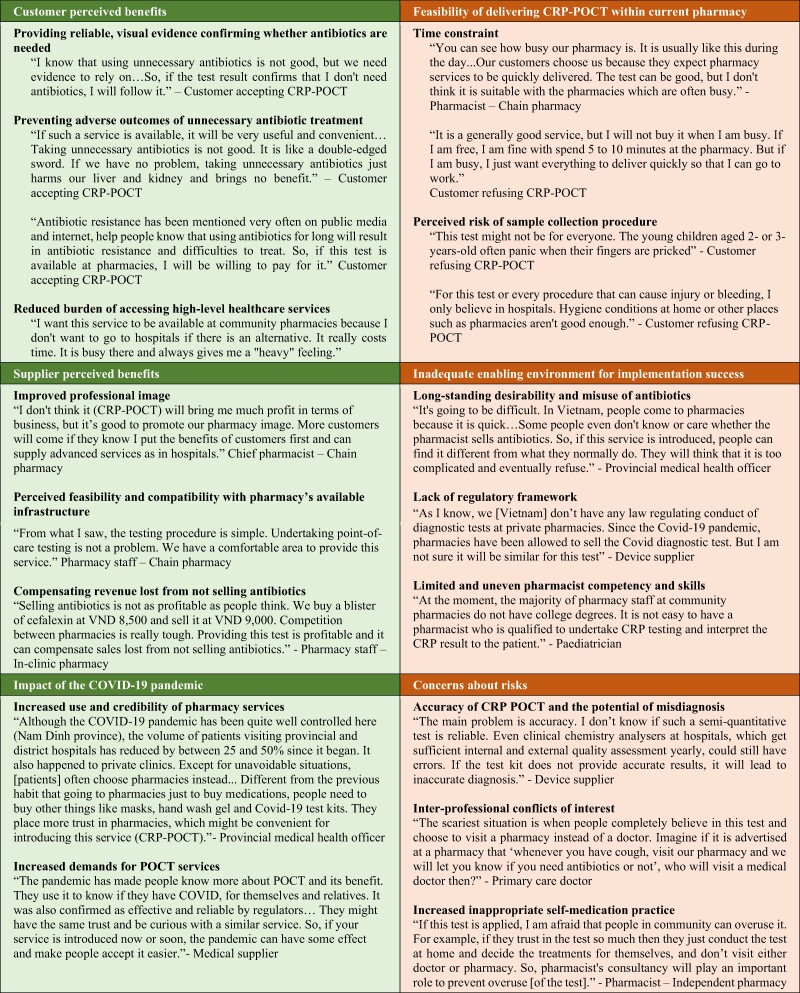

Enablers included customers’ and suppliers’ perceptions of the benefits of the CRP-POCT service. Respondents noted that implementing CRP-POCT at pharmacies would improve antibiotic targeting and welcomed convenient access to a diagnostic testing service usually provided only at hospitals. They also thought it would help prevent the adverse outcomes of unnecessary antibiotic treatment. The CRP-POCT procedure was considered easy to undertake and compatible with the current pharmacy infrastructure. It could help strengthen the professional image of the pharmacy and compensate for lost revenue from not selling antibiotics. The COVID-19 pandemic also acted as an enabler through changes in healthcare-seeking patterns. Experiences of using COVID-19 rapid test kits during the COVID-19 pandemic increased people's trust in pharmacy POCT services.

Challenges included perceptions about the feasibility of delivering CRP-POCT within current pharmacy transactions. Concerns were raised about time constraints and the risk of infection from finger-prick tests in pharmacies. There were also concerns about the lack of an enabling environment for successful implementation, including the persistent habit of overusing antibiotics in the community, the lack of a legal framework to regulate pharmacy POCT services, and the perceived limitations in pharmacist skills to correctly interpret the test. Finally, there were concerns about risks to the integrity of healthcare, such as the potential for misdiagnosis with CRP-POCT, increased inter-professional conflicts, and the promotion of inappropriate self-medication practices.

## DISCUSSION

We found that CRP-POCT to improve antibiotic targeting was well accepted by pharmacy customers, and the WTP threshold was realistic. Nearly 80% of pharmacy customers seeking treatments for ARIs were willing to pay for CRP-POCT with a mean WTP of USD$2.40. This is just under the cost of the Actim test we used (USD$2.70) but falls within the cost range of different CRP-POCT products approved in Europe and the United States (USD$1–7) [20]. Antibiotics were sold to more than 80% of patients with CRP levels below 10 mg/L, for whom they were unlikely to have been needed. More than 95% of customers who experienced CRP-POCT confirmed that it should be offered at community pharmacies.

Our qualitative findings confirmed the high acceptability of CRP-POCT. Customers perceived CRP-POCT as a visual and reliable tool to improve antibiotic targeting and minimize adverse outcomes of unnecessary antibiotic treatment. The COVID-19 pandemic likely facilitated the acceptability of POCT services at community pharmacies through the role of pharmacies in supplying and performing COVID-19 POCTs. Globally, management of the COVID-19 pandemic demonstrated the benefits of rapid tests to a wide spectrum of customers and other stakeholders and increased demand for other POCT services in pharmacies [24].

Although 96.5% of customers who experienced CRP-POCT supported its future introduction in pharmacies, only 27.7% stated that they would change their decision about antibiotic use according to the CRP result and 69.0% were unsure about how the test result would influence their decision. This was much lower than what was found in Western Australia, where 50.9% of customers changed their perception of their need for antibiotics [17]. From our qualitative findings, we believe that the community's long-standing desire for antibiotics, concerns about accuracy and the potential for misdiagnosis, and public confidence in pharmacists’ competence may contribute to customers’ reluctance to change their behavior. Although the first factors may take time to address, increasing public confidence in pharmacists’ competence should be addressed in a timely and appropriate manner for such innovative pharmacy-based interventions to be introduced to the public. In Australia, public trust in pharmacists’ competency was found to be an enabler, whereas in our study, some raised this concern. Our qualitative findings echoed other studies, with respondents raising concerns about pharmacies staffed by sellers with little or no formal pharmacy training. Introducing CRP-POCT first at large pharmacies staffed with degree-qualified pharmacists trained in the responsible use of antibiotics could help.

Customers’ belief in the role of antibiotics decreased customers’ acceptance of CRP-POCT in the quantitative component and was mentioned as a challenge for implementation in the qualitative component. This has also been observed in previous studies, in which the social belief in the ubiquitous role of antibiotics is among the major drivers of antibiotic overuse [25, 26]. This suggests that CRP-POCT introduction must be harmonized with interventions aiming to improve community awareness. The lower acceptance among customers seeking treatments for children, also confirmed by qualitative findings, demonstrates that CRP-POCT is not a one-size-fits-all approach and may need to be tailored for particular population groups.

Our analysis highlighted the importance of gaining insight into the introduction of the CRP-POCT intervention from a market access perspective, in terms of both researching customer behavior and pricing CRP-POCT. First, we found that customers who had ARI symptoms for more than 3 days were more likely to accept the service. This means that customers were only willing to pay out of pocket to understand the cause of a suspected illness if they had been bothered by the symptoms for a certain time. Second, we found that customers’ income was associated with higher WTP. This suggests that the socioeconomic context needs to be considered for correct pricing and sustainable implementation of CRP-POCT [27]. Although pharmacy owners in wealthy regions, with customers willing to pay higher prices for CRP-POCT, might find it straightforward to price the service, those in low-income regions might struggle to find a suitable price to balance customers’ WTP and profitability.

Inter-professional conflicts of interest between pharmacists and primary care doctors and the lack of a legal framework and treatment guidelines for implementing CRP-POCT at community pharmacies were raised as potential barriers to implementation. Implementation of CRP-POCT would need to be considered within the context of the local healthcare system. Recent efforts by the Vietnamese Ministry of Health to decentralize the healthcare system, decrease the burden on services at higher level facilities, and enhance the capacity of primary care settings might be an enabler to the introduction of CRP-POCT at pharmacies. Similar efforts to improve access to primary care services through pharmacies are already being rolled out in other countries, such as the Pharmacy First initiative in the United Kingdom [28]. This service will allow pharmacists to supply medicines for 7 common health conditions, including earache, sore throat, and urinary tract infections. However, concerns have been raised that this could have serious consequences for antibiotic overuse and AMR unless POCT is also used [29]. The perspectives of Vietnamese policymakers on how CRP-POCT services at pharmacies could align with decentralization efforts would be valuable, alongside insights into integrating CRP-POCT into the health system and establishing a legal framework for implementation. This study contributes important evidence on the acceptability, efficacy, and potential risks of CRP-POCT that can be taken to further discussions with policymakers in Vietnam. Evaluating the efficacy of CRP-POCT testing in pharmacies on reducing antibiotic supply without affecting patient's health outcomes would be the next step towards generating evidence required for policymakers.

Previous studies have shown that CRP is not a gold standard biomarker for the diagnosis of bacterial infections, and there is a risk of misdiagnosis when making decisions based on CRP levels alone. The development of diagnostic algorithms according to different CRP cutoffs needs to be included in future research. In addition, the use of semiquantitative CRP tests is simple and very convenient in pharmacies, but their cutoffs for decision making may not be ideal or in line with the current standard of care. In our study, 12 of 213 patients (5.6%) tested had CRP levels between 10 and 40 mg/L, which would have required further clinical judgment to determine the appropriateness of antibiotic treatment. Current standards often use the 20–30 mg/L cutoff as a lower limit for not prescribing antibiotics [30]. However, because our test kit did not have this cutoff, patients with CRP levels between 10 and 40 mg/L were still classified as unlikely to require antibiotic treatment. This may lead to misclassification. Furthermore, host-directed testing is not the only approach for POCT to guide antibiotic treatment. Pathogen-directed testing for bacterial ARI infections is not available in primary care settings in Vietnam, but future studies could consider this. However, few pathogen-directed tests are sufficiently accurate for use at point-of-care, and utility would vary widely depending on local etiologies [31, 32]. Single tests of host inflammation, such as CRP, are likely to be more cost-effective and thus more attractive to pharmacy customers who are paying out of pocket, than pathogen-directed testing where multiple tests may be required [33].

This is the first study investigating the acceptability and WTP for CRP-POCT in support of managing ARIs at community pharmacies in an LMIC setting. Compared with 2 studies in high-income countries, our study had a larger sample size and used a more standardized method to value customers’ WTP (iterative bidding game vs a single multiple-choice question to elicit WTP). It is also the first study on this topic conducted after the start of the COVID-19 pandemic, enabling this study to consider the impact of the pandemic on the acceptability of POCT services in pharmacies. A parallel cluster randomized controlled trial in Nigeria showed that the use of CRP-POCT reduced antibiotic dispensing for ARIs by 15.7% at private pharmacies. However, this study did not explore issues related to uptake or feasibility and was conducted in pharmacies where rapid diagnostic tests for malaria were also provided so that customers were already familiar with POCT services requiring finger-prick tests. Our qualitative research was more comprehensive than previous studies because we collected responses from diverse stakeholders representing those who might be involved in future service introduction rather than focusing on only 2 stakeholder (pharmacists [34]).

This study has several limitations. First, there are several factors that may affect the representativeness of our findings. The study collected data mainly from 1 region (Nam Dinh city), which limits the representativeness of our findings for other settings (rural or other regions of Vietnam). Although the proportion of pharmacies that refused to participate in our study was low (<25%), and the majority justified their refusal on the grounds of avoiding the risk of COVID-19 infection for pharmacy customers, we could not eliminate the possibility of participation bias. Conducting our studies only in registered pharmacies also limits the extrapolation of results to unregistered pharmacies, which make up an important proportion of all pharmacies in LMICs like Vietnam. We targeted registered pharmacies because they were more accessible and more suitable for the initial introduction of such an innovative intervention. As there is no published evidence on the demographic characteristics of pharmacy customers in Vietnam, we had no prior reference to check whether our study sample was representative. However, because the acceptance rate was >90%, the risk of participation bias was low and our participants may be representative of pharmacy customers in the relevant population. Second, collecting data during the COVID-19 pandemic is both a strength and a limitation. The COVID-19 pandemic might have influenced respondents’ perceptions about POCTs in pharmacies, but we do not know whether this influence will be sustained in the long term. Pharmacy customers (both in terms of patterns and behaviors) during a pandemic could be also distinctive. Third, the testing procedure was conducted by study doctors and nurses instead of pharmacists after a pharmacy transaction had already occurred, and this might not be reflective of the scenario where CRP-POCTs are done by pharmacists before a transaction and might have affected both acceptability and WTP for CRP-POCT. Further research is needed to evaluate CRP-POCT in routine pharmacy practice, where test procedures are not standardized and performed by qualified health professionals. Fourth, similar to other WTP studies using an iterative bidding game to elicit WTP, we could not eliminate the influence of starting point bias. Although we applied best practices to reduce the potential for bias by using different initial bidding points, with the range obtained from a literature review, its impact was still confirmed in our regression analysis. Finally, qualitative data analysis was conducted by 1 person (N. V. N.) with previous knowledge and experiences in pharmacy practice and health systems in Vietnam and could have been influenced by subjective opinions.

In conclusion, CRP-POCT services at community pharmacies are a feasible and well-accepted strategy to tackle the overuse of antibiotics in the community. Widespread use of POCT during the COVID-19 pandemic is likely to have bolstered support for CRP-POCT. Potential challenges that need to be addressed to successfully implement CRP-POCT include creating an enabling environment for implementation and raising awareness of benefits and risks among customers and stakeholders.

## Supplementary Data

Supplementary materials are available at *Open Forum Infectious Diseases* online. Consisting of data provided by the authors to benefit the reader, the posted materials are not copyedited and are the sole responsibility of the authors, so questions or comments should be addressed to the corresponding author.

## Supplementary Material

ofae445_Supplementary_Data

## Notes

***Author Contributions***. N. V. N. and S. O. L. were responsible for conceptualization and study design. P. B. B. and T. T. V. coordinated study activities across the sites with N. V. N., T. Q. P., and S. O. L. undertook supervision and local support. V. T. K. and A. T. L. provided oversight. N. V. N. accessed, cleaned, managed, and verified the data. N. V. N. led the data analysis with supervision from S. O. L. N. V. N. drafted the manuscript with supervision from S. O. L. All authors contributed to the final version of the manuscript and approved the submission. The corresponding author had final responsibility to submit for publication.

***Financial support*.** This work was supported by the Medical Research Council, UK, grant number MR/S001964/1.

***Data sharing statement***. Data may be available according to the data-sharing policy of the relevant partners contributing to this study upon request to the corresponding author.

## References

[1] Nguyen KV , Thi Do NT, Chandna A, et al Antibiotic use and resistance in emerging economies: a situation analysis for Viet Nam. BMC Public Health 2013; 13:1158. 24325208 10.1186/1471-2458-13-1158 PMC4116647

[2] Torumkuney D , Kundu S, Vu GV, et al Country data on AMR in Vietnam in the context of community-acquired respiratory tract infections: links between antibiotic susceptibility, local and international antibiotic prescribing guidelines, access to medicines and clinical outcome. J Antimicrob Chemother 2022; 77(Supplement_1):i26–34. 36065731 10.1093/jac/dkac214 PMC9445855

[3] Nguyen TTP , Do TX, Nguyen HA, et al A national survey of dispensing practice and customer knowledge on antibiotic use in Vietnam and the implications. Antibiotics (Basel) 2022; 11:1091. 36009960 10.3390/antibiotics11081091 PMC9405246

[4] Nga TT , Chuc NT, Hoa NP, et al Antibiotic sales in rural and urban pharmacies in northern Vietnam: an observational study. BMC Pharmacol Toxicol 2014; 15:6. 24555709 10.1186/2050-6511-15-6 PMC3946644

[5] Jamshed S , Padzil F, Shamsudin SH, et al Antibiotic stewardship in community pharmacies: a scoping review. Pharmacy (Basel) 2018; 6:92. 30142902 10.3390/pharmacy6030092 PMC6163858

[6] Afari-Asiedu S , Abdulai MA, Tostmann A, et al Interventions to improve dispensing of antibiotics at the community level in low and middle income countries: a systematic review. J Glob Antimicrob Resist 2022; 29:259–74. 35342021 10.1016/j.jgar.2022.03.009

[7] Lam TT , Dang DA, Tran HH, et al What are the most effective community-based antimicrobial stewardship interventions in low- and middle-income countries? A narrative review. J Antimicrob Chemother 2021; 76:1117–29. 33491090 10.1093/jac/dkaa556

[8] Chan JTN , Nguyen V, Tran TN, et al Point-of-care testing in private pharmacy and drug retail settings: a narrative review. BMC Infect Dis 2023; 23:551. 37612636 10.1186/s12879-023-08480-w PMC10463283

[9] Collaborators GBDLRI . Estimates of the global, regional, and national morbidity, mortality, and aetiologies of lower respiratory infections in 195 countries, 1990–2016: a systematic analysis for the Global Burden of Disease Study 2016. Lancet Infect Dis 2018; 18:1191–210. 30243584 10.1016/S1473-3099(18)30310-4 PMC6202443

[10] Finley CR , Chan DS, Garrison S, et al What are the most common conditions in primary care? Systematic review. Can Fam Physician 2018; 64:832–40. 30429181 PMC6234945

[11] Martínez-González NA , Keizer E, Plate A, et al Point-of-care C-reactive protein testing to reduce antibiotic prescribing for respiratory tract infections in primary care: systematic review and meta-analysis of randomised controlled trials. Antibiotics (Basel) 2020; 9:610. 32948060 10.3390/antibiotics9090610 PMC7559694

[12] Cals JWL , Butler CC, Hopstaken RM, Hood K, Dinant G-J. Effect of point of care testing for C reactive protein and training in communication skills on antibiotic use in lower respiratory tract infections: cluster randomised trial. BMJ 2009; 338:b1374. 19416992 10.1136/bmj.b1374 PMC2677640

[13] Do NT , Ta NT, Tran NT, et al Point-of-care C-reactive protein testing to reduce inappropriate use of antibiotics for non-severe acute respiratory infections in Vietnamese primary health care: a randomised controlled trial. Lancet Glob Health 2016; 4:e633–41. 27495137 10.1016/S2214-109X(16)30142-5 PMC4985565

[14] Do NTT , Vu TVD, Greer RC, et al Implementation of point-of-care testing of C-reactive protein concentrations to improve antibiotic targeting in respiratory illness in Vietnamese primary care: a pragmatic cluster-randomised controlled trial. Lancet Infect Dis 2023; 23:1085–94. 37230105 10.1016/S1473-3099(23)00125-1

[15] Matthes A , Wolf F, Wilde E, Bleidorn J, Markwart R. Point-of-care measurement of C-reactive protein promotes de-escalation of treatment decisions and strengthens the perceived clinical confidence of physicians in out-of-hours outpatient emergency medical services. BMJ Open 2023; 13:e069453. 10.1136/bmjopen-2022-069453 PMC10163444 37147098

[16] Wakeman M , Cork T, Watwood D. Point-of-care C-reactive protein testing in community pharmacy to deliver appropriate interventions in respiratory tract infections. Clin Pharm 2018; 10:149–53.

[17] Sim TF , Chalmers L, Czarniak P, et al Point-of-care C-reactive protein testing to support the management of respiratory tract infections in community pharmacy: a feasibility study. Res Social Adm Pharm 2021; 17:1719–26. 33500197 10.1016/j.sapharm.2021.01.004

[18] Onwunduba A , Ekwunife O, Onyilogwu E. Impact of point-of-care c-reactive protein testing intervention on non-prescription dispensing of antibiotics for respiratory tract infections in private community pharmacies in Nigeria: a cluster randomized controlled trial. Int J Infect Dis 2022;127:137–43. 36509332 10.1016/j.ijid.2022.12.006 PMC9876806

[19] Palinkas LA , Horwitz SM, Green CA, Wisdom JP, Duan N, Hoagwood K. Purposeful sampling for qualitative data collection and analysis in mixed method implementation research. Adm Policy Ment Health 2015; 42:533–44. 24193818 10.1007/s10488-013-0528-y PMC4012002

[20] Thi Thuy Do N , Greer RC, Lubell Y, et al Implementation of C-reactive protein point of care testing to improve antibiotic targeting in respiratory illness in Vietnamese primary care (ICAT): a study protocol for a cluster randomised controlled trial. BMJ Open 2020; 10:e040977. 10.1136/bmjopen-2020-040977 PMC7759760 33361164

[21] Hansen KS , Pedrazzoli D, Mbonye A, et al Willingness-to-pay for a rapid malaria diagnostic test and artemisinin-based combination therapy from private drug shops in Mukono District, Uganda. Health Policy Plan 2013; 28:185–96. 22589226 10.1093/heapol/czs048 PMC3584993

[22] Steigenberger C , Flatscher-Thoeni M, Siebert U, Leiter AM. Determinants of willingness to pay for health services: a systematic review of contingent valuation studies. Eur J Health Econ 2022; 23:1455–82. 35166973 10.1007/s10198-022-01437-x PMC8853086

[23] Braun V , Clarke V. Using thematic analysis in psychology. Qual Res Psychol 2006; 3:77–101.

[24] Karako K , Song P, Chen Y, Tang W. Increasing demand for point-of-care testing and the potential to incorporate the internet of medical things in an integrated health management system. Biosci Trends 2022; 16:4–6. 35197419 10.5582/bst.2022.01074

[25] McKinn S , Trinh DH, Drabarek D, et al Drivers of antibiotic use in Vietnam: implications for designing community interventions. BMJ Glob Health 2021; 6:e005875. 10.1136/bmjgh-2021-005875 PMC8278923 34257138

[26] Nguyen HH , Ho DP, Vu TLH, et al “I can make more from selling medicine when breaking the rules”—understanding the antibiotic supply network in a rural community in Viet Nam. BMC Public Health 2019; 19:1560. 31771536 10.1186/s12889-019-7812-z PMC6880519

[27] Bala MV , Mauskopf JA, Wood LL. Willingness to pay as a measure of health benefits. Pharmacoeconomics 1999; 15:9–18. 10345161 10.2165/00019053-199915010-00002

[28] Wickware C . Pharmacy First will be introduced in England in 2023, says government. 2023. Available at: https://pharmaceutical-journal.com/article/news/pharmacy-first-will-be-introduced-in-england-in-2023-says-government. Accessed 20 October 2023.

[29] Bachmann T , Beardmore R, Charani E, et al Scientists’ AMR warning over government's new ‘pharmacy-first’ policy—letter. 2023. Available at: https://www.telegraph.co.uk/global-health/science-and-disease/scientists-amr-warning-government-pharmacy-first-policy/. Accessed 20 October 2023.

[30] van Vugt SF , Broekhuizen BD, Lammens C, et al Use of serum C reactive protein and procalcitonin concentrations in addition to symptoms and signs to predict pneumonia in patients presenting to primary care with acute cough: diagnostic study. BMJ 2013; 346:f2450. 23633005 10.1136/bmj.f2450 PMC3639712

[31] Aston SJ . The role of rapid diagnostic tests in managing adults with pneumonia in low-resource settings. Pneumonia 2014; 5(Suppl 1):8–17. 26290807 10.15172/pneu.2014.5/444 PMC4538792

[32] Orda U , Mitra B, Orda S, et al Point of care testing for group A streptococci in patients presenting with pharyngitis will improve appropriate antibiotic prescription. Emerg Med Australas 2016; 28(2):199–204. 26934845 10.1111/1742-6723.12567

[33] Lubell Y , Althaus T, Blacksell SD, et al Modelling the impact and cost-effectiveness of biomarker tests as compared with pathogen-specific diagnostics in the management of undifferentiated fever in remote tropical settings. PLoS One 2016; 11:e0152420. 27027303 10.1371/journal.pone.0152420 PMC4814092

[34] Czarniak P , Chalmers L, Hughes J, et al Point-of-care C-reactive protein testing service for respiratory tract infections in community pharmacy: a qualitative study of service uptake and experience of pharmacists. Int J Clin Pharm 2022; 44:466–79. 35088232 10.1007/s11096-021-01368-2 PMC8794609

