# Supplementary material for: Understanding Acceptability and Willingness-to-pay for a C-reactive Protein Point-of-care Testing Service to Improve Antibiotic Dispensing for Respiratory Infections in Vietnamese Pharmacies: A Mixed-methods Study
Source: Open Forum Infect Dis. 2024 Aug 2;11(8):ofae445. doi: 10.1093/ofid/ofae445 (PMC11347944; doi:10.1093/ofid/ofae445)
Supplement: ofae445_Supplementary_Data [file ofae445_supplementary_data.zip › Sup1. Study design.pptx]

## Slide 1
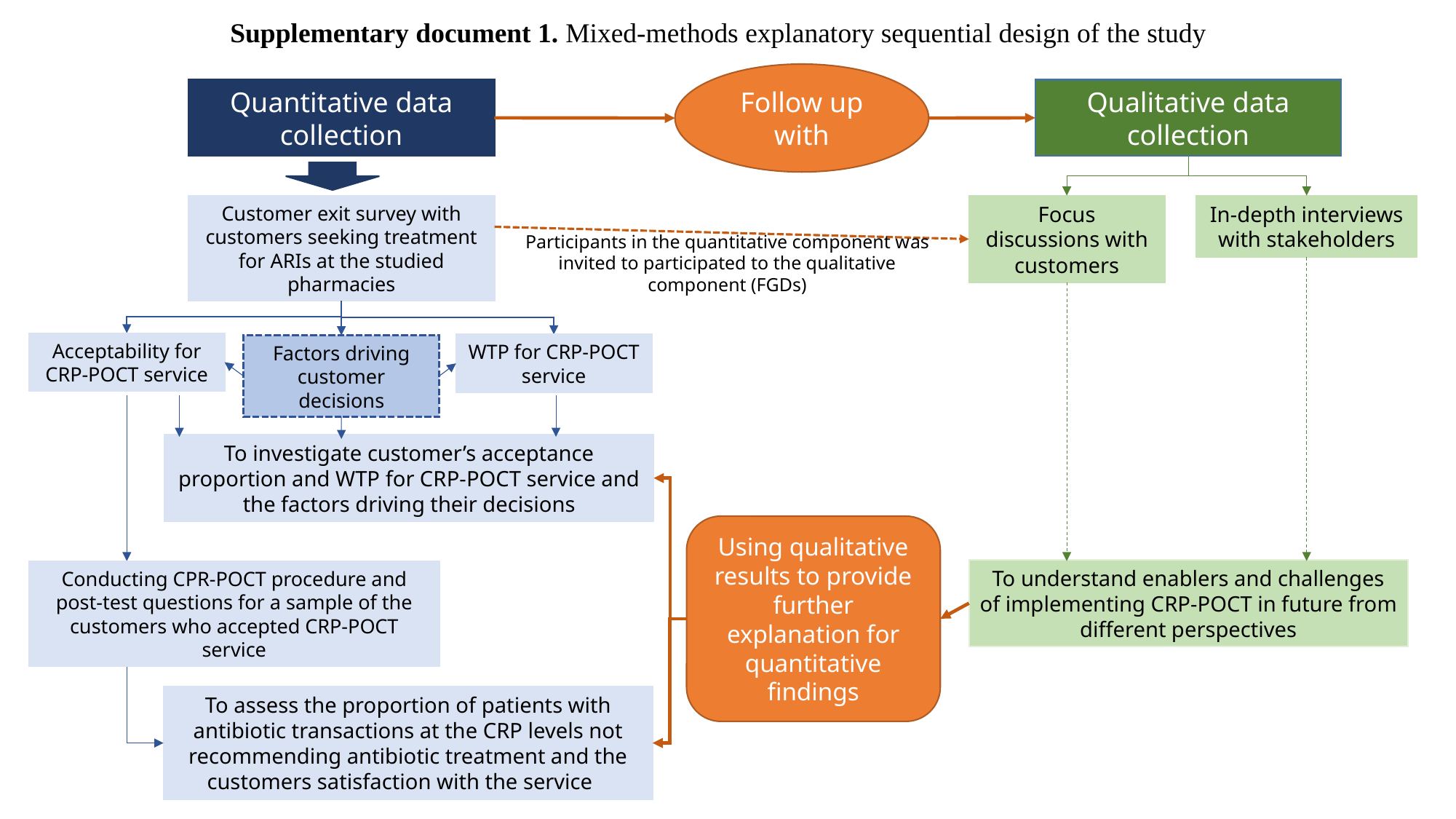

Supplementary document 1. Mixed-methods explanatory sequential design of the study
Follow up with
Qualitative data collection
Quantitative data collection
Focus discussions with customers
In-depth interviews with stakeholders
Customer exit survey with customers seeking treatment for ARIs at the studied pharmacies
Participants in the quantitative component was invited to participated to the qualitative component (FGDs)
Acceptability for CRP-POCT service
WTP for CRP-POCT service
Factors driving customer decisions
To investigate customer’s acceptance proportion and WTP for CRP-POCT service and the factors driving their decisions
Using qualitative results to provide further explanation for quantitative findings
To understand enablers and challenges of implementing CRP-POCT in future from different perspectives
Conducting CPR-POCT procedure and post-test questions for a sample of the customers who accepted CRP-POCT service
To assess the proportion of patients with antibiotic transactions at the CRP levels not recommending antibiotic treatment and the customers satisfaction with the service
