# Supplementary material for: Understanding Acceptability and Willingness-to-pay for a C-reactive Protein Point-of-care Testing Service to Improve Antibiotic Dispensing for Respiratory Infections in Vietnamese Pharmacies: A Mixed-methods Study
Source: Open Forum Infect Dis. 2024 Aug 2;11(8):ofae445. doi: 10.1093/ofid/ofae445 (PMC11347944; doi:10.1093/ofid/ofae445)
Supplement: ofae445_Supplementary_Data [file ofae445_supplementary_data.zip › Sup2. Sample size calculation.docx]

**Supplementary document 2.** Sample size calculation

We calculated that a sample size of 500 customer exit interviews (25 pharmacies with 20 interviews per pharmacy) would be needed to estimate the mean willingness-to-pay for a CRP-POCT with a 95% confidence interval of 15%, assuming a mean of US$ 0.80, a standard deviation of US$ 0.70 and an intra-cluster correlation of 0.15. Our sample size calculation for WTP estimation was similar to a previous study investigating the WTP for a rapid malaria diagnostic test from private drug shops in Uganda.^1^ Allowing for around 5% refusal rate and ensuring sufficient data collection for data analyses, the estimated overall sample size was 520.

A sample size of 520 was also sufficient to estimate the proportion of accepting CRP-POCT with 95% confidence interval of 5%, assuming that around 50% of participants would accept CRP-POCT (required sample size of 385 according to the Cochrane’s formula). The assumption of 50% is recommended for studies lacking estimates to calculate sample size because it requires the highest sample size.^2^ It is suitable for my study because there has been no previously published evidence quantifying the acceptability for the out-of-pocket CRP-POCT service, or similar rapid testing services, at pharmacies.

We estimated that half of participants would be willing to pay for CRP-POCT (n=260). These patients would be offered CRP-POCT. The sample size of 260 was sufficient to estimate the proportion of unnecessary antibiotic transactions (based on the CRP test results) with 95% confidence interval of 5%, assuming that more than 80% of participants were supplied antibiotics unnecessarily (required sample size of 246 according to Cochrane’s formula).^2^ The assumption of 80% was based on previously published evidence on inappropriate antibiotic supply at community pharmacies in Vietnam.^3,4^

REFERENCE

1. Hansen KS, Pedrazzoli D, Mbonye A, et al. Willingness-to-pay for a rapid malaria diagnostic test and artemisinin-based combination therapy from private drug shops in Mukono District, Uganda. *Health Policy Plan* 2013; **28**(2): 185-96.

2. Barlett JE, Kotrlik JW, Higgins CC. Organizational research: Determining appropriate sample size in survey research. *Information Technology, Learning, and Performance Journal* 2001; **19**(1): 43-50.

3. Nga do TT, Chuc NT, Hoa NP, et al. Antibiotic sales in rural and urban pharmacies in northern Vietnam: an observational study. *BMC Pharmacol Toxicol* 2014; **15**: 6.

4. Zawahir S, Le HTT, Nguyen TA, et al. Inappropriate supply of antibiotics for common viral infections by community pharmacies in Vietnam: A standardised patient survey. *Lancet Reg Health West Pac* 2022; **23**: 100447.
