# Supplementary material for: Understanding Acceptability and Willingness-to-pay for a C-reactive Protein Point-of-care Testing Service to Improve Antibiotic Dispensing for Respiratory Infections in Vietnamese Pharmacies: A Mixed-methods Study
Source: Open Forum Infect Dis. 2024 Aug 2;11(8):ofae445. doi: 10.1093/ofid/ofae445 (PMC11347944; doi:10.1093/ofid/ofae445)
Supplement: ofae445_Supplementary_Data [file ofae445_supplementary_data.zip › Sup3. Details of eligibility criteria.docx]

**Supplementary document 3.** Details of eligibility criteria

The eligible participants needed to meet all of the inclusion criteria and none of the exclusion criteria including:

**Inclusion criteria:**

- aged ≥18 years.
- Pharmacy customer seeking treatment for mild ARI for self or relatives.
- at least one focal or systemic ARI symptom including cough, runny nose, sore throat, shortness of breath, wheezing or chest pain/discomfort lasting no longer than 2 weeks prior to the pharmacy encounter.
- written informed consent to participate.

**Exclusion criteria:**

- no access to telephone.
- Patients diagnosed with a severe respiratory infection by a doctor prior to the pharmacy visit.
- Patients who had already taken antibiotics or were not able to recall the kinds of treatment they took prior to customer’s pharmacy encounter.
- Patients who had baseline conditions that might bias CRP-POCT-based diagnosis including pregnancy, liver disease, immunocompromised (HIV, undergoing chemotherapy, long term usage) or chronic inflammatory (gout).
