# Supplementary material for: Understanding Acceptability and Willingness-to-pay for a C-reactive Protein Point-of-care Testing Service to Improve Antibiotic Dispensing for Respiratory Infections in Vietnamese Pharmacies: A Mixed-methods Study
Source: Open Forum Infect Dis. 2024 Aug 2;11(8):ofae445. doi: 10.1093/ofid/ofae445 (PMC11347944; doi:10.1093/ofid/ofae445)
Supplement: ofae445_Supplementary_Data [file ofae445_supplementary_data.zip › Sup4. Customer exit questionnaire .docx]

**Supplementary document 4**

Encounter customer exit questionnaire to investigate acceptability and willingness-to-pay for CRP-POCT service at community pharmacies

|  | Screening questions | Answer |
| --- | --- | --- |
| 1 | How old are you? | \|__\|__\| years  *(If under 18 years STOP interview)* |
| 2 | What sex are you? | 1 = Male  2 = Female |
| 3 | Who did you come to buy medicine for today? | 1 = Self  2 = Spouse/partner  3 = Child  4 = Parent/parent-in-law  5 = Friend  6 = Animal *(STOP interview)*  7 = Other (specify ______________) |
| 4 | How old is the person you are buying medicine for | \|__\|__\| years  *(If under 1 year put* \|__\|__\|*months)* |
| 5 | What sex are they? | 1 = Male  2 = Female |
| 6 | What is the main problem you/this person is suffering from today that prompted you to seek treatment?  *Circle all that apply* | 1 = Cough or cold  2 = Blocked or runny nose  3 = Fever  4 = Chills  5 = Myalgia  6 = Sore throat  7 = Sinusitis  8 = Head ache  9 = Ear ache  10 = Rapid or difficult breathing related to a problem in the chest  *(STOP interview and refer to healthcare provider)*  11 = Other (specify _____________)  *(If only Other STOP interview)* |
| 7 | How long have you/they been suffering from these symptoms? | \|__\|__\| days \|__\|__\| weeks |
| 8 | Is the person who is sick today… | 1 = Currently pregnant?  2 = Immunocompromised (e.g. HIV, long-term corticosteroid use)?  3 = Suffering from liver disease  *(If any circled STOP interview)*  9 = None of the above |
| 9 | If we need to reach you for further follow-up, do you have a phone that we can contact you on, and do you agree to be contacted? If yes to both, what is the number. | 1 = Yes (#_____________________)  2 = No |
|  | Background questions | Answer |
| 10 | What do you think the best treatments are for the symptoms you/the person who us sick is suffering from today?  *Circle all mentioned* | 1 = Antibiotics  2 = Analgesics/antipyretics  3 = Antiallergics  3 = Traditional medicine or herbs  4 = Vitamins and tonics  5 = Bed rest and fluids  6 = Other (specify ______________) |
| 11 | Does the person who is sick today have a health insurance card? | 1 = Yes  2 = No  9 = Don’t know |
| 12 | Did the person who is sick today seek care from anyone/anywhere else before coming here? | 1 = Yes  2 = No à Skip to 20 |
| 13 | Where did the patient go? | 1 = Commune health centre  2 = District polyclinic  3 = District hospital  4 = Provincial hospital  5 = Private clinic/hospital  6 = Other (specify ______________) |
| 14 | When did the patient go? | 1 = Today  2 = Within the last 3 days  3 = Within the last week  4 = Other (specify______________) |
| 15 | What was the diagnosis? | 1 = Non-severe respiratory illness  2 = Other (specify ______________) |
| 16 | Did they receive any prescription | 1 = Yes  2 = No à Skip to 18 |
| 17 | What was the prescription? |  |
| 18 | Why did you choose to go to the pharmacy for medications today instead of going to the clinic/hospital?  *Circle all mentioned* | 1 = Medications not available  2 = Medications not covered by health insurance  3 = Medications are more expensive  4 = Convenience  5 = Already went to clinic/hospital but wasn’t prescribed any medications  6 = Medications from clinic/hospital/covered by health insurance were not effective  7 = No diagnostic tests are required at the pharmacy  8 = Other (specify ______________) |
| 19 | Did the pharmacist suggest any medications (apart from your prescribed medications)? | 1 = Yes (specify________________)  2 = No |
| 20` | Did you request any medications (apart from your prescribed medications)? | 1 = Yes (specify________________)  2 = No |
| 21 | What kind of medication did you get today for the person who is sick? | 1 = Antibiotics (specify__________)  2 = Analgesics/antipyretics  3 = Antiallergics  3 = Traditional medicine or herbs  4 = Vitamins and tonics  5 = Other (specify______________) |
| 22 | If you don’t mind, can I see it?  *(Write down the names of all medications obtained)* |  |
| 23 | What was the total cost of the treatment you bought at the pharmacy today? | \|__\|__\|__\|__\|__\|__\|__\| VND |
| 24 | If you bought antibiotics, what was the cost of only the antibiotics? | \|__\|__\|__\|__\|__\|__\|__\| VND |
| 25 | Has anyone else in your household been suffering from similar symptoms/illness in the last 2 weeks? | 1 = Yes  2 = No |
| 26 | Have you/the person who is sick ever had any bad experiences (side effects) with antibiotic use | 1 = Yes  2 = No à Skip to 28 |
| 27 | If yes, please mention  *Circle all mentioned* | 1 = Allergy  2 = Diarrhoea  3 = Hospitalized due to side effects  4 = Others |
|  | Factors affecting willingness-to-pay | Answer |
| 28 | What is the highest level of school you have completed? | 1 = None  2 = Primary  3 = Secondary  4 = College or university  5 = Graduate school |
| 29 | What is your marital status? | 1 = Married/co-habiting  2 = Single/not in a relationship |
| 30 | What is your occupation? | 1 = Farmer  2 = Labourer  3 = Craftsman  4 = Factory worker  5 = Health worker  6 = Office worker  7 = Student  8 = Unemployed  9 = Other (specify ______________) |
| 31 | Might you share with me information on your average monthly household income within the last 12 months | \|__\|__\|__\|__\|__\|__\|__\|__\|__\|__\| VND |
| 32 | How many people usually live in your house? | \|__\|__\| people |
| 33 | What is the first healthcare provider that you go to when you or your family members have minor health issues (e.g. cough, cold, blocked or runny nose, chills, myalgia, sore throat, sinusitis, head ache, ear ache)? | 1 = Commune health centre  2 = District polyclinic/hospital  4 = Private clinic/hospital  5 = Community pharmacy  6 = Other (specify ______________) |
| 34 | How many times have you been to a pharmacy to seek healthcare recommendations/services for any reason in the last 6-months? | 1 = At least once every week  2 = Every 1-2 weeks  3 = About once a month (6 times)  4 = 3-5 times in the last 6 months  5 = 1-2 times in the last 6 months  6 = Haven’t been in the last 6 months |
| 35 | How do you usually travel to the pharmacy? | 1 = Walking  2 = Bicycle  3 = Motorbike  4 = Car  5 = Other (Specify______________) |
| 36 | How long does it take you to travel to the pharmacy by this method? | 1 = <15 minutes  2 = 15-29 minutes  3 = 30=59 minutes  4 = 60 minutes or more |
|  | Patients’ satisfaction with pharmacy services |  |
| 37 | On a scale of 1 to 10), are you satisfied with the following condition of the pharmacy | Score (1 to 10, with 1= Not at all satisfied and 10= Extremely satisfied) |
| 38 | The location of the pharmacy | \|__\|__\| |
| 39 | The cleanliness and hygienic condition | \|__\|__\| |
| 40 | The comfort and privacy at the waiting and counseling area | \|__\|__\| |
| 41 | The comfort and safety of the parking facility | \|__\|__\| |
| 42 | The appropriate and safe storage of medicines | \|__\|__\| |
| 43 | The availability of medicines and other health products that you need | \|__\|__\| |
| 44 | The quality and/or origins of medicines and other health products that you need | \|__\|__\| |
| 45 | The prices of medicines and other health products that you need | \|__\|__\| |
| 46 | The knowledge of the counseling pharmacist | \|__\|__\| |
| 47 | The attitude of the counselling pharmacist | \|__\|__\| |
| 48 | The age/ experiences of the counselling pharmacist | \|__\|__\| |
| 49 | The communication method and skills of the counselling pharmacist | \|__\|__\| |
| 50 | The counselling time that the pharmacist spent with you | \|__\|__\| |
| 51 | The necessary instructions and warnings of the pharmacist on your treatment (dosage regimen, side effects and potential risks, diet and physical activities during treatment) | \|__\|__\| |
| 52 | The non-paid services/facilities such as blood pressure measuring, weighing machine, home deliver or any other? | \|__\|__\| |
| 53 | The paid medicines/supplements and other health services beyond your prescription that the pharmacist counsel you to buy? | \|__\|__\| |
| 54 | In general, how much are you satisfied with the pharmacy care services at community pharmacies in your city? | \|__\|__\| |
| 55 | Can you explain your answer? |  |
|  | CRP scenario and permission | Answer |
| 56 | I will now provide you with some information on antibiotics and a diagnostic test called C-reactive protein. This test is used in hospitals, but we would like to see whether it could help customers make decisions about their treatment at pharmacies.  When your respiratory system is infected by either a virus or bacteria, you will typically suffer from a similar range of symptoms. However, antibiotics are only effective at treating bacterial infection, and do not work for most coughs and colds, which are almost always caused by viruses. If antibiotics are prescribed for a viral infection, they do not cure the disease or lessen the symptoms. Using antibiotics when they are not needed can cause great harm to both the user, family members and the community at large. It will both increase treatment cost unnecessarily and increase the risk of developing bacteria that don’t respond to antibiotic treatment (i.e. antibiotic resistance).  Now, there is a test called a CRP rapid test. It measures a chemical in the blood that is produced by your body when you get an infection. If this chemical level is low it is unlikely you have a bacterial infection, and this can tell you that you don’t need antibiotics. This test requires a simple finger prick test. The results will be available after 90 seconds. The results of this test today will not be used to provide a clinical assessment or treatment for your illness, but to demonstrate how the test works. The CRP test strip has some lines to indicate levels of an infection marker in your blood. If the CRP is lower than the 10mg/L line, you are unlikely to have a bacterial infection, and you can decide whether you want to see a healthcare provider or not. If your CRP level is above the 10mg/L line, you may still not have a bacterial infection, and you may get better without any medications. However, if you are worried at any time or the symptoms get worse you should go and see a healthcare provider.  **CRP test permission inquiry**  Based on the information you were told, are you willing to undertake the CRP test now after our guidance? If you are not the sick person, are you willing to bring them to the pharmacy for a CRP test now? | 1 = Yes à follow CRP testing procedure  2 = No à follow CRP scenario procedure and explain the reason for your decision |
| 57 | **CRP testing procedure:**  - Sanitize  - Finger prick to obtain capillary blood (10 µl)  - Analyzed using the ACTIM CRP Rapid Test (Medix, Biochemica).  - Results will be available after 90 seconds | CRP test result  1 = <10 mg/L  2 = ³10 £40 mg/L  3 = >40 mg/L |
| 58 | **CRP scenario procedure:**  - Describe the CRP procedure to the participant |  |
|  | Bidding game |  |
| 59 | If the pharmacy now offers a CRP testing service, would you be willing to pay … VND (initial bidding price) for it to show you if your illness is unlikely to benefit from treatment with antibiotics?  If yes, are you willing to pay … + 20,000 VND? (repeating the bidding game following the guidance in the Figure 4-1. | \|__\|__\|__\|__\|__\|__\|__\| VND |
| 60 | You said you would be willing to pay …………… VND for the test. If you also had to buy medicine, how would this affect the amount you are willing to pay for the test? | 1 = The same  2 = Different (Explain and give new amount ______________________  \|__\|__\|__\|__\|__\|__\|__\| VND) |
|  | Open-ended questions | Answer |
| 61 | What are your overall thoughts about the CRP test? *Probe: How useful is it? How practical is it? How acceptable is it?* |  |
| 62 | If you had done this test before you bought medicine/treatment today, how would this have affected your decisions about medicine/treatment today? *Probe: Would you still have bought antibiotics? Would you have gone to see a healthcare provider?* |  |
| 63 | Would you like to see tests like this available in your local pharmacy? Explain. |  |
| 64 | Who would you feel comfortable doing this test?  *Circle all that apply* | 1 = Buy a kit to take home and do it yourself  2 = Pharmacist  3 = Pharmacy staff  4 = Would prefer to have it done by a qualified health-worker (nurse or doctor)  5 = Other |
| 65 | Can you tell me more about your reason for this answer? |  |
| 66 | How would having this test available in pharmacies affect your treatment choice for mild illnesses? |  |
