# Supplementary material for: Understanding Acceptability and Willingness-to-pay for a C-reactive Protein Point-of-care Testing Service to Improve Antibiotic Dispensing for Respiratory Infections in Vietnamese Pharmacies: A Mixed-methods Study
Source: Open Forum Infect Dis. 2024 Aug 2;11(8):ofae445. doi: 10.1093/ofid/ofae445 (PMC11347944; doi:10.1093/ofid/ofae445)
Supplement: ofae445_Supplementary_Data [file ofae445_supplementary_data.zip › Sup5. Bidding game.docx]

**Supplementary document 5.** Figure illustrates our iterative bidding procedure to elicit customer’s WTP. The bidding process was developed based on the previously studies on WTP of healthcare interventions or non-market services.^1-3^


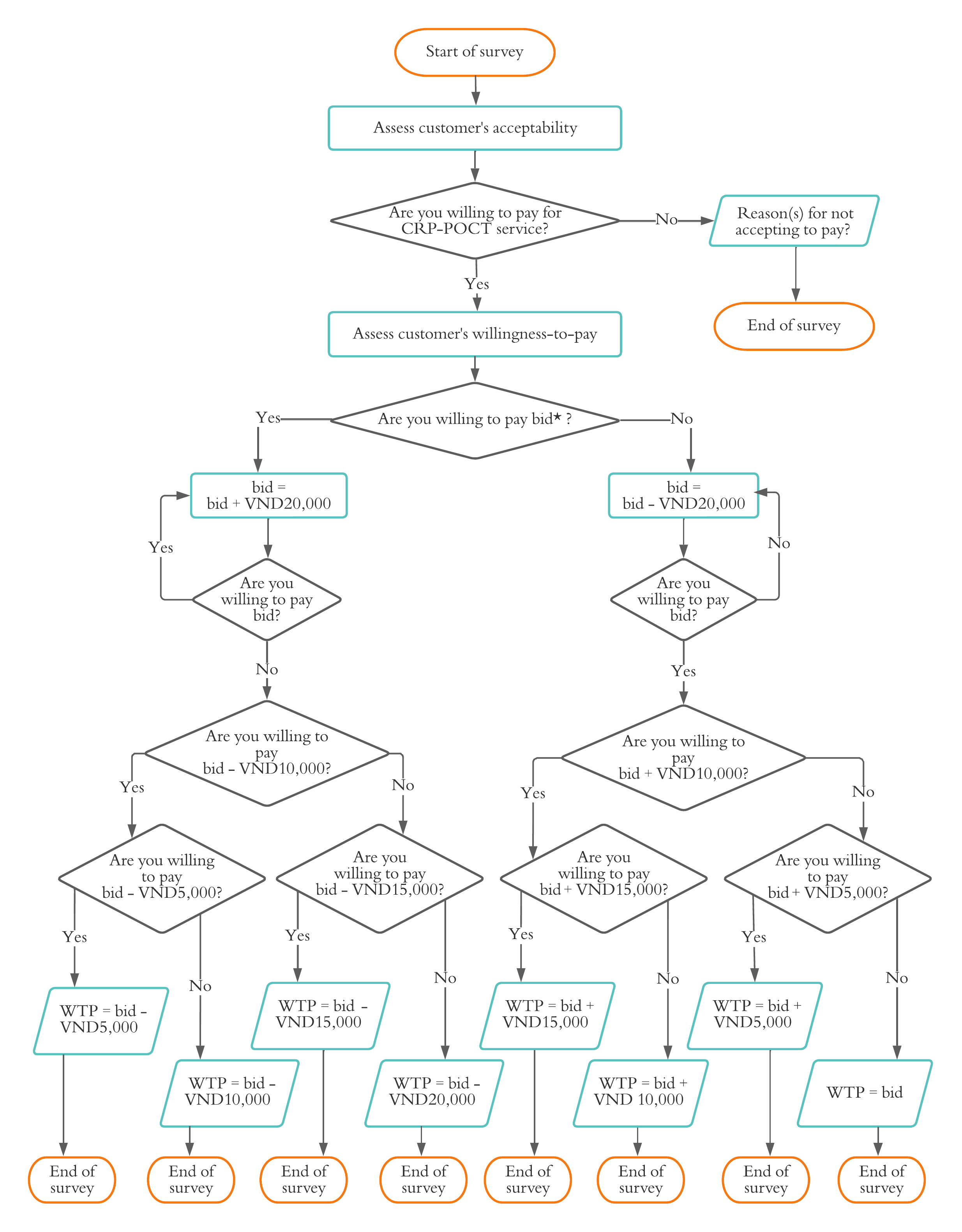


**: Initial price bid was randomly assigned among 4 values: VND 15,000, 50,000, 100,000 and 150,000 ($US 0.7, 2.2, 4.3 and 6.5, respectively. The VND was transferred into the US$ currency using the 2021 World Bank exchange rate [*[*https://data.worldbank.org/indicator/PA.NUS.FCRF*](https://data.worldbank.org/indicator/PA.NUS.FCRF)*]: US$1 ~ VND23,159.78).*

The monetary currency used in the bidding game was Vietnamese Dong (VND) (US$1 ~ VND23,159.78 according to the 2021 World Bank exchange rate (<https://data.worldbank.org/indicator/PA.NUS.FCRF>). All of the respondents accepting CRP-POCT service were invited to elicit WTP valuation. The respondents rejecting the service were asked what drove their decision through an open-ended question. In each WTP survey, the interviewer started WTP valuation by asking the respondent if they are willing to pay for CRP-POCT service at an initial price bid. This initial price bid was randomly assigned among VND 15,000, 50,000, 100,000 and 150,000 ($US 0.7, 2.2, 4.3 and 6.5, respectively). The initial bid of each interview was hidden in sealed envelopes which was only opened at the end of the survey to start the bidding game. This prevented the interviewer from subjectively selecting an initial bid that they assumed suited the respondent's economic status, leading to information bias. I used four different initial bids to consider impacts of starting point bias. The range between VND 15,000 to 150,000 was determined through our literature review of published studies investigating the unit cost of providing CRP-POCT in other healthcare settings and customer’s WTP for other pharmacy rapid tests for infectious diagnosis.^4-9^ I assumed that this range was suitable to explore WTP values ranging from VND 10,000 to VND 250,000, equivalent to approximately $US 0.5 to $US10.

If the respondent answered “yes” to the first WTP valuation question “are you willing to pay for CRP-POCT service at initial price bid, they would be asked the question again with a higher monetary amount by VND 20,000. If they continued to answer "yes", these questions would be iterated with amounts increasing by VND 20,000 until the respondent answered "no". The interviewer would then decrease the nearest bid by VND 10,000 and ask if the respondent would accept this new bid. In the final interaction, the offered bid would slightly increase again by VND 5,000 if the respondent said "yes" or decreased by VND 5,000 if the respondent said "no", and the customer's WTP would be determined. On the contrary, if the respondent indicated “no” for the first WTP valuation question, the interviewer would offer a lower price bid (bid – VND 20,000) and continued asking until the respondent answered “yes”. The process would be iterated similarly as above until the customer's WTP was elicited.

There were two exceptions. First, the respondent started by a "No" answer and continued it until the bid decreased to an amount lower than VND 10,000. In this situation, the respondent would be classified into not accepting the service and we would ask why they only accepted the service at such a low price. Second, the initial bid was randomly VND 15,000 and the respondent answered "No" for the first question. At the time, the bidding would be proceeded by decreasing the bid to VND 10,000 and the respondent was asked if they would accept this new price. If they said "yes", their WTP would value VND, 10,000. If they said "no", they would be classified into not accepting. Similar as above, the respondent was asked why s/he was only willing to pay for the service at such a low price.

REFERENCE

1. Frew EJ, Wolstenholme JL, Whynes DK. Comparing willingness-to-pay: bidding game format versus open-ended and payment scale formats. *Health Policy* 2004; **68**(3): 289-98.

2. Onwujekwe O. Searching for a better willingness to pay elicitation method in rural Nigeria: the binary question with follow-up method versus the bidding game technique. *Health Econ* 2001; **10**(2): 147-58.

3. McNamee P, Ternent L, Gbangou A, Newlands D. A game of two halves? Incentive incompatibility, starting point bias and the bidding game contingent valuation method. *Health Econ* 2010; **19**(1): 75-87.

4. Oppong R, Jit M, Smith RD, et al. Cost-effectiveness of point-of-care C-reactive protein testing to inform antibiotic prescribing decisions. *Br J Gen Pract* 2013; **63**(612): e465-71.

5. Hunter R. Cost-effectiveness of point-of-care C-reactive protein tests for respiratory tract infection in primary care in England. *Adv Ther* 2015; **32**(1): 69-85.

6. Holmes EAF, Harris SD, Hughes A, Craine N, Hughes DA. Cost-Effectiveness Analysis of the Use of Point-of-Care C-Reactive Protein Testing to Reduce Antibiotic Prescribing in Primary Care. *Antibiotics (Basel)* 2018; **7**(4).

7. Uzochukwu BSC, Onwujekwe OE, Uguru NP, Ughasoro MD, Ezeoke OP. Willingness to pay for rapid diagnostic tests for the diagnosis and treatment of malaria in southeast Nigeria: ex post and ex ante. *International Journal for Equity in Health* 2010; **9**(1): 1.

8. Hansen KS, Pedrazzoli D, Mbonye A, et al. Willingness-to-pay for a rapid malaria diagnostic test and artemisinin-based combination therapy from private drug shops in Mukono District, Uganda. *Health Policy Plan* 2013; **28**(2): 185-96.

9. Lubell Y, Do NTT, Nguyen KV, et al. C-reactive protein point of care testing in the management of acute respiratory infections in the Vietnamese primary healthcare setting – a cost benefit analysis. *Antimicrobial Resistance & Infection Control* 2018; **7**(1): 119.
