# Supplementary material for: Understanding Acceptability and Willingness-to-pay for a C-reactive Protein Point-of-care Testing Service to Improve Antibiotic Dispensing for Respiratory Infections in Vietnamese Pharmacies: A Mixed-methods Study
Source: Open Forum Infect Dis. 2024 Aug 2;11(8):ofae445. doi: 10.1093/ofid/ofae445 (PMC11347944; doi:10.1093/ofid/ofae445)
Supplement: ofae445_Supplementary_Data [file ofae445_supplementary_data.zip › Sup6. Qualitative Interview guide.docx]

**Supplementary document 6.** Interview guides for in-depth interviews and focus group discussions on enablers and challenges for the implementation of CRP-POCT service at community pharmacies

| Ice breaker (5 mins) |
| --- |
| Informed consent (5 mins) |
| Participants’ information (2 mins) |
| Participant ID  Age  Occupation  Type of qualification  Number of years since qualification |
| Interview part 1 (3 mins) |
| 1. To start, describe your job and how long have you been working? 2. How do you see the role of community pharmacists within the healthcare system? 3. What are your opinions about the current cooperation between community pharmacists and other healthcare professionals (e.g, primary care doctors)? Do you think it could be improved? If yes, how could it be improved? |
| Interview part 2 (10 mins) |
| Now I would like to talk with you about antibiotic dispensing at community pharmacies. We know that there are some issues in the community with antibiotic practice and AMR, not everywhere but in some cases.   1. What do you think are the major drivers of poor/uninformed antibiotics prescribing and dispensing practices in the communities in which you work? 2. [if not discussed as part of q4] How often do people come to community pharmacies to ask for antibiotics? Do they often come with a valid prescription? How often do they come without a prescription, tell their or another patient’s symptoms, and ask for antibiotics? 3. [if not discussed as part of q4] Do pharmacists still supply antibiotics for the patients mentioned above? What are the causes of these inappropriate practices? *(Probe: lack of accessible primary care doctors, economic pressures on pharmacists and/or patients, pressures on pharmacists to maintain customers, lack of appropriate trainings, etc)* 4. Do you think these practices (from q4) cause any impact for these patients or within the wider community? If yes, what impact? 5. Are there any interventions that have been implemented to improve dispensing practices? If yes, explain.  - (Probe :Who has implemented them? Have they been effective (no, temporary or permanent)? If ineffective, why have they been ineffective? How could they be improved?)  1. What do you all think are good strategies to improve these practices? 2. Is there any group of pharmacists that have different attitudes towards antibiotic supplies for patients? (younger pharmacists, licensed pharmacists, pharmacists working in a pharmacy which are regularly audited by the government, pharmacies have participated in studies, etc)? What are the attitudes? Why do they have different attitudes? (better education, previous experiences of audits, etc) 3. Is there any group of patients that have different attitudes towards their need for antibiotics and their right to request antibiotics? *(younger patients, pregnant women, patients with medical history of medication adverse effects, etc)*? Why do they have different attitudes? *(better education, previous experiences of side effects, etc)* |
| Interview part 3 (20 mins) |
| Now I would like you to introduce a tool to help community pharmacists to know whether a patient really needs an antibiotic. It is a test called a CRP rapid test. It measures a chemical in the blood that is produced by the body when you get an infection (C-reactive protein). If this CRP level is low (below 10mg/L) it is unlikely the customer has a bacterial infection, and this can guide your decisions about prescription and dispensing of antibiotics. 75% of patients attending primary care clinics for mild respiratory illness in Ha Noi had CRP below 10mg/L. Those with CRP levels above 10mg/L may still not have a bacterial infection, but the test cannot rule it out. This test requires a simple finger prick test. The results will be available after 90 seconds.   1. What do you think about the value of this test? Do you think it could be used to tackle the current issues in inappropriate antibiotic dispensing in community pharmacies? 2. Do you think this test could be used in community pharmacies? Which are the pharmacies that you think this test could be used in? Which are the pharmacies that you think this test will be difficult to apply? (*Probe: pharmacies with licensed pharmacists, larger pharmacies)?* Why? 3. Is there any issue that makes you hesitate about the test? In your opinion, who/what could help to tackle these issues?  - Focus on: - Issues regard to pharmacists (sufficient trainings on implement the diagnosed test on patients?), patients (increased treatment cost), and primary care doctors (conflict of interest with pharmacists on patient care)  1. Do you think this test will change the pharmacy services in particular and primary care services? If yes, which way? (Probe: business implications, how to maintain patient satisfaction, and selling of other drugs for symptomatic relief, better/worse cooperation between pharmacists and primary care doctors) 2. In your opinion, if this test is widely applied, what are the positive and negative outcomes that this might bring to community health? 3. Besides this, are there interventions that you suggest that could improve antibiotic dispensing in community pharmacies? |
