# Supplementary material for: Understanding Acceptability and Willingness-to-pay for a C-reactive Protein Point-of-care Testing Service to Improve Antibiotic Dispensing for Respiratory Infections in Vietnamese Pharmacies: A Mixed-methods Study
Source: Open Forum Infect Dis. 2024 Aug 2;11(8):ofae445. doi: 10.1093/ofid/ofae445 (PMC11347944; doi:10.1093/ofid/ofae445)
Supplement: ofae445_Supplementary_Data [file ofae445_supplementary_data.zip › Sup7. Pharmacy and customer recruitment procedures.docx]

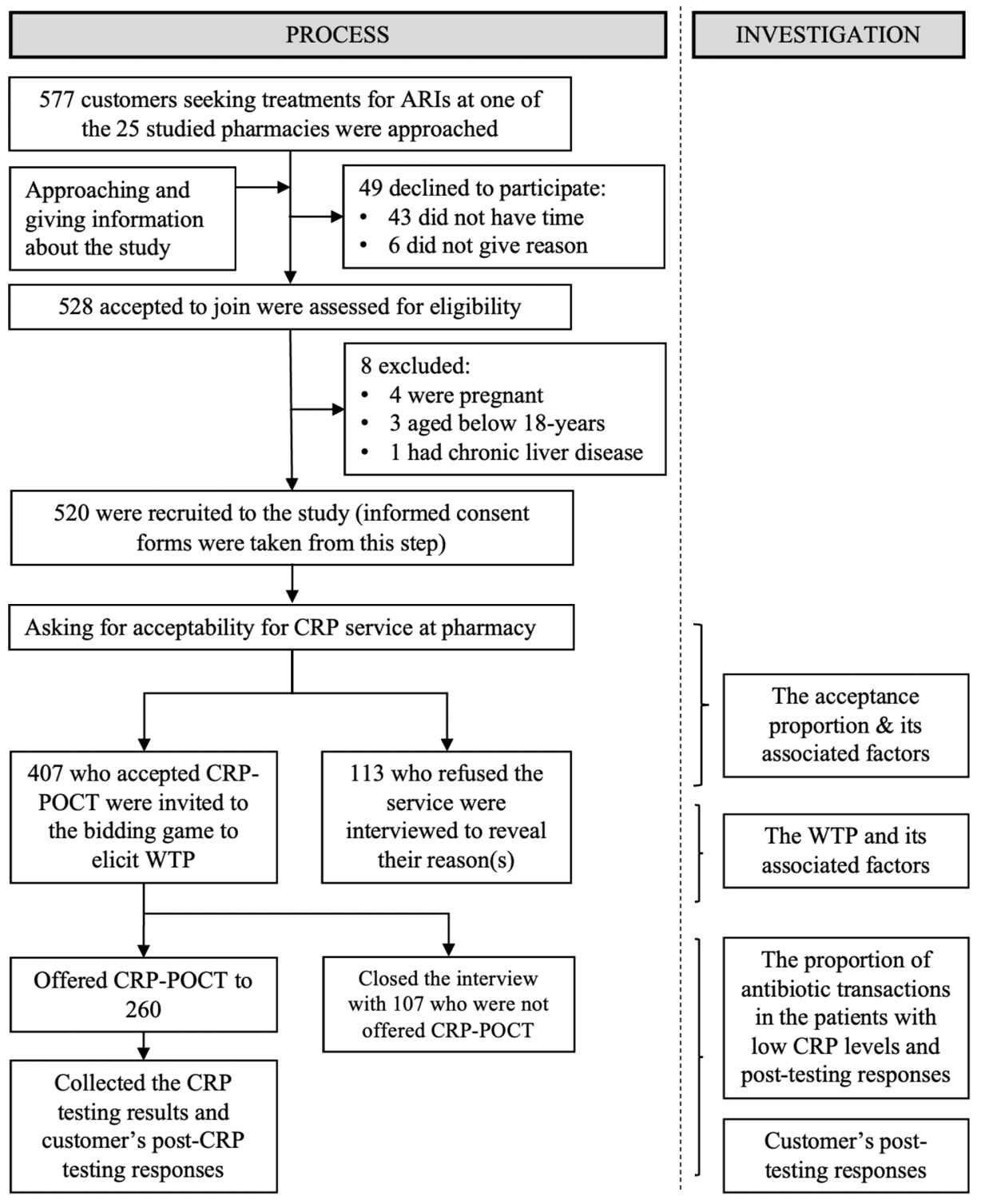


**Supplementary document 7.** Participant recruitment and assessment process.

*Informed consent forms (ICF) were obtained from all of the customers (520 in total) in compliance with the Good Clinical Practice's standards. For the cases in which a customer seeked treatments for another patient (relative) and the patient was brought to the pharmacy to experience CRP-POCT, ICF was also obtained from the patient (above 18 years old) or the patient’s legally authorized representative (if the patient below 18 years old)*

We obtained a list of the pharmacies registered by the Nam Dinh provincial department of health. From this list, we defined all of the pharmacies located in Nam Dinh city (74 in total) and contacted pharmacy owners via phone to ask if they were willing to participate in our study. 55 pharmacy owners responded to our phone calls and 42 of them (76.4% or 42/55) accepted to join. Among 13 pharmacy owners who refused to join our study, 8 were afraid of risks of COVID-19 transmission due to long interactions between interviewers and customers and 5 did not give a reason. 25 pharmacies were randomly selected from these 42 pharmacies.

At these 25 pharmacies, we approached in total 577 customers seeking treatment for ARIs and invited them to join the study. 528 accepted to join, corresponding to a participation proportion of 91.5%. The majority of the customers (87.8% or 43/49) who declined to join this study said it was due to lack of time. The study interviewers screened these 528 patients against the eligibility criteria using the first section of the questionnaire. Eight customers, including four pregnant women, three customers aged below 18-years and one having chronic liver disease were excluded according to the study protocol.

We obtained conscent and interviewed 520 customers to investigate the proportion accepting the CRP-POCT service and determinants for the acceptance. 403 customers who accepted the service joined a bidding game to elicit the WTP for the CRP-POCT and its determinants. Of these 403 customers, 260 were offered CRP-POCT. We collected data on their CRP testing results and post-test responses to investigate the proportion of antibiotic transactions among patients with low CRP levels and customer’s post-test perceptions towards CRP-POCT.
