# Supplementary material for: Understanding Acceptability and Willingness-to-pay for a C-reactive Protein Point-of-care Testing Service to Improve Antibiotic Dispensing for Respiratory Infections in Vietnamese Pharmacies: A Mixed-methods Study
Source: Open Forum Infect Dis. 2024 Aug 2;11(8):ofae445. doi: 10.1093/ofid/ofae445 (PMC11347944; doi:10.1093/ofid/ofae445)
Supplement: ofae445_Supplementary_Data [file ofae445_supplementary_data.zip › Sup8. Sentitivity analyses for WTP.docx]

**Supplementary document 8.** Factors affecting WTP for CRP-POCT testing service at community pharmacy to guide antibiotic treatment in patients with ARIs.

|  | Model 1 (main) | | Model 2 | |
| --- | --- | --- | --- | --- |
|  | β (SE) | p | β (SE) | p |
| Age | -0.004 (0.002) | 0.075† | -0.004 (0.004) | 0.326 |
| Sex  Male (ref)  Female | -  -0.025 (0.058) | 0.504 | -  -0.067 (0.110) | 0.543 |
| Education class  Preliminary school (ref)  High school  University/college or higher degree | -  0.060 (0.081)  0.044 (0.082) | 0.537  0.471 | -  0.137 (0.155)  0.154 (0.156) | 0.377  0.323 |
| Income groups  < VND 3,000,000 (< $US 129.5) (ref)  VND 3,000,000 – 5,000,000 ($US 129.5 - 259.1)  > VND 5,000,000 (> $US 259.1) | -  0.266 (0.081)  0.232 (0.079) | 0.001^**^  0.004^**^ | -  0.370 (0.154)  0.330 (0.152) | 0.017^*^  0.029^*^ |
| Customer’s perception on the role of antibiotics to the patient’s condition  Antibiotic treatment is not needed (ref)  Antibiotic treatment is needed | -  0.004 (0.067) | 0.950 | -  0.010 (0.128) | 0.940 |
| Antibiotic transaction  Yes  No (ref) | 0.124 (0.075)  - | 0.100 | 0.207 (0.142)  - | 0.146 |
| Child patient  Yes  No (ref) | 0.109 (0.118)  - | 0.353 | 0.123 (0.224)  - | 0.583 |
| Seeing doctor prior pharmacy visit  Yes  No (ref) | -0.064 (0.144)  - | 0.657 | -0.124 (0.276)  - | 0.653 |
| Duration of illness  ≤3 days (ref)  >3 days | -  0.029 (0.070) | 0.667 | -  0.014 (0.128) | 0.915 |
| Type of pharmacy visited  Privately owned pharmacy (ref)  Retailed pharmacy chain  In-hospital/in-clinic pharmacy | -  0.156 (0.127)  -0.116 (0.136) | 0.230  0.400 | -  0.393 (0.246)  -0.126 (0.262) | 0.123  0.634 |
| Cost of total drug treatment | 0.134 (0.061) | 0.027^*^ | 0.311 (0.115) | 0.007^**^ |
| Degree of satisfaction to pharmacy service | 0.002 (0.042) | 0.954 | -0.038 (0.080) | 0.636 |
| Initial bidding price | 0.059 (0.120) | <0.001^***^ | 0.116 (0.023) | <0.001^***^ |
| Number of observations ^a^  Constant  R^2^ | 392  0.128  0.160 | | 392  1.522  0.163 | |

*Notes. ***p < .001, **p < .01, *p < .05, †p < .10. β, beta-coefficients; SE, standard error; WTP, willingness to pay; CRP-POCT, C-reactive protein point-of-care testing.*

*^a:^ Observations with missing values were dropped (listwise deletion). More specifically, listwise deletion means that observations with missing data were deleted and only the reduced sample of complete observations (complete case analysis) is analysed. In our analyses, 392 among the total of 407 paritipants (96.3% or 392/407) who accepted CRP-POCT were included.*

*Two mixed effects linear regression model were performed with similar independent variables and different mathematical forms of dependent variable (WTP). Model 1 is the main model, with participants' WTP transformed into log values. The log transformation was made because the distributions of WTP values were right skewed while the distributions of their log transformed values were more normalized. Model 2 was performed for a sensitivity analysis to assess the robustness of the main model, with WTP kept as their original values. In both models, fixed effects included age, sex, education class, income groups, customer’s perception on the role of antibiotic treatment, status of purchasing antibiotics, child patients, status of seeing doctor prior pharmacy visit, duration of illness, type of pharmacy, cost of total drug treatment, degree of satisfaction to pharmacy service and initial bidding price (to consider starting point bias). Random effect included pharmacy ID.*
